# Physicochemical, Functional, and Nutraceutical Properties of Eggplant Flours Obtained by Different Drying Methods

**DOI:** 10.3390/molecules23123210

**Published:** 2018-12-05

**Authors:** Jenny R. Rodriguez-Jimenez, Carlos A. Amaya-Guerra, Juan G. Baez-Gonzalez, Carlos Aguilera-Gonzalez, Vania Urias-Orona, Guillermo Nino-Medina

**Affiliations:** 1 Facultad de Ciencias Biologicas, Universidad Autonoma de Nuevo Leon, Ave. Universidad S/N, Cd. Universitaria, 66450 San Nicolas de los Garza, Mexico; ing.rodriguezjimenez@hotmail.com (J.R.R.-J.); baezjuan@yahoo.com.mx (J.G.B.-G.); carlos.aguileragn@uanl.edu.mx (C.A.-G.); 2 Laboratorio de Quimica y de Alimentos, Facultad de Salud Publica y Nutricion, Universidad Autonoma de Nuevo Leon, Col. Mitras Centro, C.P. 64460 Monterrey, Nuevo Leon, Mexico; vania.uriaso@uanl.mx; 3 Laboratorio de Quimica y Bioquimica, Facultad de Agronomia, Universidad Autonoma de Nuevo Leon, Francisco Villa S/N, Col. Ex-Hacienda El Canada, C.P. 66050 General Escobedo, Nuevo Leon, Mexico

**Keywords:** eggplant, flour, phenolics, antioxidant activity, functional ingredient

## Abstract

The importance of consuming functional foods has led the food industry to look for alternative sources of ingredients of natural origin. Eggplants are a type of vegetable that is valued for its content in phytochemical compounds and it is due to the fact that this research is conducted towards the development of eggplant flour as a proposal to be used as a functional ingredient in the food industry. In this study, the eggplant fruits were divided into four groups, based on the drying method and the equipment used: Minced, drying oven (T1); sliced, drying oven (T2); sliced and frozen, drying tunnel (T3); and sliced, drying tunnel (T4). All the eggplant flours showed the same trend regarding their antioxidant capacity and phenolic content in the order T2 > T4 > T1 > T3. The freezing of eggplant was found to have a negative effect on functional and antioxidant properties. With respect to their nutritional composition, the flours did not change in their crude fiber, protein, and fat contents. In general terms, the T2 flour is a potential ingredient for the preparation of foods with functional properties since it is rich in phenolic compounds and antioxidants.

## 1. Introduction

In recent years, the food industry has focused its efforts in the development of new products with properties that not only provide the necessary nutrients for human food, but also help prevent diseases related to nutrition such as diabetes, obesity, hypertension, and cardiovascular complications. It has been found that there is a significant correlation between the regular intake of phytochemicals and the prevention of these lifestyle-related diseases [1]. Antioxidants have attracted great attention as possible agents to prevent and treat diseases related to oxidative stress [2]. The antioxidants used by the food industry can be either from natural sources or from a synthetic origin (such as butylated hydroxytoluene and butylated hydroxyanisole). The latter has been found to be potentially carcinogenic and toxic [3]. Consequently, a niche in the food industry is opened to replace the existing synthetic antioxidants with those of natural origin found in fruits and vegetables, which are mainly vitamins and polyphenols [2].

Eggplant is an economically important vegetable crop from the tropical and subtropical zones of the world [4]. This crop produces fruit of different colors, sizes, and shapes [5]. Eggplant is a valued vegetable for its composition in phytochemicals considered as nutraceuticals [6], in particular, polyphenols and dietary fiber [4].

In Mexico, eggplant production was 172,112 tons in 2016. It is mostly exported to the United States as this vegetable is not commonly consumed domestically [7] due to a lack of information regarding its preparation and characteristics. Eggplant has a non-climacteric pattern of respiration, which leads to a short shelf life despite being harvested in immature stages of development [8]. Therefore, the use of eggplant is suggested as a flour with high nutritional value, which can also be used as an antioxidant of natural origin. Therefore, the objective of this work is to evaluate the physicochemical, functional, and nutraceutical properties of eggplant flour as a proposed functional ingredient.

## 2. Results and Discussion

### 2.1. Eggplant Flour Samples

The eggplant flour produced was labelled as T1 (eggplant minced and dried at 45 °C–50 °C in a drying oven), T2 (sliced eggplant dried at 45 °C–50 °C in a drying oven), T3 (sliced eggplant was frozen and dried at 40 °C–45 °C in a tunnel dryer), and T4 (sliced eggplant dried at 40 °C–45 °C in a tunnel dryer). Eggplant is a vegetable with a high percentage of water (approximately 90%), which allows microorganisms and biochemical reactions to deteriorate, thus reducing its shelf life. In general, eggplant is a difficult vegetable to dehydrate due to its high percentage of water, which implies long drying times. With the use of the drying tunnel, the drying time was reduced from 48 h (drying oven) to 16 h (70% reduction in efficiency), showing that dehydration is faster when the air speed increases [9] and the speed of drying at high temperature decreases due to the hardening phenomenon [10,11,12].

### 2.2. Proximal Chemical Analysis 

The results of the nutritional composition of the eggplant flours are shown in Table 1. Eggplant flour has low values for moisture content (1.5% to 8.5%), below the Mexican standard (NOM-247-SSA1-2008) of 15% [13]. The T2 sample had the highest moisture content, while the T4 sample had the lowest moisture content. The moisture content obtained in this study was lower than other results (7.7% to 9.45%) reported from different types of *Solanum melongena*, dried in the same range (45 °C to 50 °C) of temperature [4,14].

Flour having a moisture content of 9% to 10% is suitable for extended shelf life [15] since a lower moisture content in flour shows a better storage stability. The range of the average ash content determined among the four eggplant flours was 6.47%–7.31%, and it was similar to the eggplant ash content of other investigations treated under the same drying temperature conditions [4,14], compared to the ash content obtained from different types of eggplants (0.48%–1%), and 4.93%–13.7% (dry base) [16]; the drying treatment allows the concentration of the eggplant nutrients. Regarding the determination of proteins, the results obtained fell in a small range of 12.55%–12.77%. The results of this study are in accordance with the USDA database [17]. They have reported that protein content for fresh eggplant was 0.98% (12.73% in dry basic). Various types (Indian, Thai, Chinese, and white) of eggplants dried at the same temperature produced similar protein contents (12%–15%) [4] to the results obtained in this study.

The average fat content (1.75%) of the flour in this study was higher than that reported by Nino-Medina et al. [16] in fresh eggplant (Chinese, Philippine, Thai, Hindu and American types), with obtained values between 0.3% and 0.4% (dry base). Uthumporn et al. [4] found levels of 0.88% to 5.18% in different types (Indian, Thai, Chinese, and white) of eggplant flour; the lowest values were for flour samples made at 50 °C. Carbohydrates contents for the samples were between 57% and 65%. The result of the present investigation is similar in the amount of carbohydrates contained in the eggplant flour mentioned before, which were in the range of 62%–68%. The main soluble sugars were glucose and fructose [18]. They reported starch content between 1.43% and 2.38% in fresh eggplant. Eggplant flour contained a lower amount of carbohydrates and moisture compared with wheat flour, yet it had more fiber.

### 2.3. Physicochemical Parameters

The pH and titratable acidity are analytically determined in separate ways, and each has its own particular impact on food quality [19]. The pH is a good predictor of the ability of a microorganism to grow in a specific food, while the titratable acidity is a good predictor of the impact of acid content on the flavor of food [20]. On the other hand, color is the first notable characteristic of a food and often predetermines our expectations. Natural and synthetic colors play several roles in foods and consumers use the color as a way to identify a food and also as a way to judge the quality of a food [21].

With the exception of the titratable acidity and b* chromatic property, in which statistical differences were not observed (*p* > 0.05), other physicochemical parameters showed statistical differences (*p* < 0.05) between eggplant flour samples (Table 2). The values of pH of eggplant flours were slightly acidic and ranged from 3.89 to 4.14, while titratable acidity values were low ranging from 0.46% to 0.47%. In addition, chromatic values were from 52.50 to 64.60, 4.55 to 9.65, 20.15 to 21.65, 21.09 to 23.60, and 65.98 to 77.54 in L*, a*, b*, C*, and *h*, respectively. All the eggplant flours had a “mostly desaturated dark orange” color. However, the color of the treatments 1 and 2 can be classified as a “pale brown”, while the treatments the color of the treatments 3 and 4 can be classified as “clear brown”; the main difference among these two colors tonalities is due mainly to the L* value.

The cause of this color difference is attributed to the enzymatic browning of vegetable tissue, which is one of the main causes of loss of quality in food drying. The color values corresponding to the T1 and T2 samples show the effect caused by the Maillard reaction in eggplant during the drying process due to the formation of brown complex polymers (melanins) [22]. The T1 and T2 samples are more affected by this phenomenon due to the long drying times in the drying oven. The sample T3 shows a color similar to the aforementioned samples due to the damage by the low temperatures to which it was subjected before drying.

There is no literature available for comparison with the current report as there are no studies on the evaluation of chromatic properties of eggplant flour; however, flours obtained from other vegetables through similar methods to the ones used in this study have been previously reported. In this regard, Noor and Komathi [23] obtained flour from peeled pumpkin pulp and unpeeled pumpkin pulp. Their process for production of flour consisted in soaking the pumpkin pulps in a 0.1% sodium methabisulphite for 30 min; after that, the pulps were washed, sliced, and dried overnight at 60 °C. The chromatic properties of the obtained flours were 63.45, 15.68, 53.83, 56.07, and 73.76 for peeled pumpkin pulp flour and 64.93, 13.53, 49.45, 51.27, and 74.70 for unpeeled pumpkin pulp flour in L*, a*, b*, C* and *h* chromatic parameters. On the other hand, Que et al. [24] (2007) also obtained flour from pumpkin through hot air-drying procedures. In this study, the pumpkin flesh was cut into slices and hot air-dried at 70 °C for 54 h. Both products were ground and sieved using a 60 mesh screen (250 μm). The chromatic properties of the obtained flours were 80.15, 13.43, 48.63, 50.45, and 74.56 for freeze-dried flour, and 61.83, 11.12, 41.87, 43.32, and 75.13 for hot air-dried flour in L*, a*, b*, C* and *h* chromatic parameters.

All the chromatic parameters obtained in the studies mentioned above were higher than the chromatic properties of our eggplant flours; this could be mainly attributed to the fact that pumpkin has different chemical and physical characteristics from eggplant. Another important fact that produces a lower L* value in eggplant in contrast to pumpkin is the high concentration of phenolics in the eggplant skin (anthocyanins) and pulp (phenolic acids), which are oxidized by an enzymatic mechanism once they are sliced, and also to the non-enzymatic browning due to the heat treatment used in the production of the flour.

### 2.4. Functional Properties

The water holding capacity (WHC) of the samples was between 1.2 to 2 g water/g flour (Table 3). Sample T4 (2.08 g water/g flour) had the highest amount of WHC and T1 (1.28 g water/g flour) had the lowest values. Similar values were found in frozen-dried flour from soy beans (1.8 g water/g flour) and pumpkin flour (1.5–2.5 g water/g flour) dried at 60 °C [23,25]. The capacity to absorb water is considered a functional property of proteins, fundamental in viscous foods such as sauces, soups, baked goods, and doughs, products where a good protein-water interaction is required [26]. Different protein structure and different hydrophilic carbohydrates contribute to the variation in WHC of flours [27,28]. This agrees with the result of Chen and et al. [29], study which reported that high WHC of fruit fibers is linked to the high pectin content of the fruits. The WHC aids modification of texture and viscosity in formulated food.

The oil holding capacity (OHC) differed significantly (*p* ≤ 0.05) among T1, T2, T3, and T4 (Table 3). *Treculia africana* seed flour, prepared at 100 °C, parboiled and dried (55 °C, 24 h) had an OHC in the range of 1.14–1.3 g oil/g for flour [30,31] and the flour from soy beans (1.93 g oil/g flour) [25] had lower values than the eggplant samples. However, the *Canavalia ensiformis* flour (3.15 g oil/g flour) [32] had similar values to these results. This high oil holding capacity can be attributed to the high levels of nonpolar residues protein molecules [32]. On the other hand, the heat treatment increases the absorption of oil [31]. This is an increase attributed to the dissociation and denaturation of proteins by heat. The T4 and T2 treatments have a greater water/oil retention capacity than the T1 and T3 samples; these changes in the retention capacity can be attributed to the modification of the physical structure of the food. Methods of food processing such as freezing and mincing can affect protein conformation and hydrophobicity [33,34].

For the emulsification capacity (EC), T1 (25%) had the lowest value with respect to the T2, T3, and T4 (Table 3), as Yu et al. [34] suggests, food processing methods affect protein conformation and hydrophobicity. The mincing process was the most probable reason for the lower EC of the T1 sample. Emulsification capacity is considered as an index of the ability of proteins or peptides to adsorb on the new created surface, delaying coalescence [35]. According to Kinsella et al. and Sathe et al. [36,37], the emulsifying capacity of proteins tend to decrease as protein concentration is increased; nevertheless, it was the opposite in this study.

In short, these functional properties verify the application of this flour as an ingredient in the formulation of a food, as the physical-chemical characteristics define the behavior of proteins, carbohydrates, and fibers in the processed food.

### 2.5. Total Phenols Content (TPC)

Polyphenols are a large group of phytochemicals that are considered responsible for the health benefits associated with fruits and vegetables [38]. Plant polyphenols can scavenge free radicals due to their chemical structure. The total phenols content (TPC) was markedly higher in samples T2 and T4 (Table 4), while it was lower for samples T3 and T1 (4183 and 8211 mg chlorogenic acid/kg flour, respectively). Similar data were reported [39] in the juice from 31 eggplant varieties (commercial varieties, landraces, and hybrids between the landraces) that were in the range of 5450 to 10,480 (mg chlorogenic acid/kg of sample). It was found that eggplant displays an important intraspecific variation for the composition traits studied, and in some cases, there are considerable differences among the varietal types.

Nino-Medina et al. [16] report similar results in their report based on a study in frozen, dried eggplant from different varieties (Chinese, Philippine, American, Hindu, and Thai); the total phenols content ranged from 15,120 to 20,490 (mg chlorogenic acid/kg of sample). The results obtained in this study were higher than the results of fresh eggplant by Nisha et al. [38] on different eggplant varieties that reported to contain between 490 to 1070 (mg gallic acid equivalents/kg of sample) and 570 to 650 (mg chlorogenic acid/kg of sample) for Black Beauty and Violetta Lunga varieties [40]. The low value of the T3 sample is due to the freezing before the dehydration; freezing reduces the original value of the food up to 80% due to the increase in water activity [41]. This has a greater effect than the mincing the sample, as it was in the case of the T1 sample.

### 2.6. Total Flavonoids Content (TFC)

The total flavonoids content of different eggplant flours is shown in Table 4. TFC content follows the order T2 > T4 > T1 > T3. According to these results, a significant difference was found between the pre-drying treatments (mincing, slicing, and slicing/freezing), where the T3 sample was the most affected by the freezing treatment of eggplant slices before drying, showing the same behavior as the TPC. The decrease in TFC level in the flour subjected to pre-drying treatments (mincing and slicing/freezing) could occur because part of the anthocyanin was degraded during these treatments. Ninfali et al. [40] report a total flavonoid content between 257 and 284 mg caffeic acid equivalents/kg of the sample in fresh Black Beauty and Violetta Lunga eggplant varieties. Uthumporn et al. [4] found a range between 9090 and 29,180 mg catechin equivalent/kg, but their report was based on a study of eggplant flour dried at same temperature range as the one used in this study.

### 2.7. Total Catechins Content (TCC)

The content of total catechins was maintained in the range of 1240 to 3022 (mg catechins/kg flour), which reveals a significant difference between tannin contents of the four eggplant flour extracts (*p* < 0.05). Indeed, the T1 extract presented the highest level among the four flour samples. Alkurd et al. [42] obtained 4137 mg tannic acid equivalents/kg from eggplant extract whole fruit, while Boulekbache et al. [43] obtained 42.6 mg tannic acid equivalents/kg from eggplant peels extract. The mincing of the eggplants before the drying process had a significant effect on the TCC in comparison with the other treatments after drying the eggplant.

### 2.8. Total Anthocyanins

The total anthocyanins content of different eggplant flours is shown in Table 4. The results were in the range of 230 a 1612 (mgC3GE/kg Flour DW) and follows the order T1 > T2 > T4 > T3. Anthocyanins results obtained in this study were similar to those reported by Nino et al. [16] in different eggplant types of Chinese (1287 mgC3GE/kg of eggplant), Philippine (1610 mgC3GE/kg of eggplant), American (1234 mgC3GE/kg of eggplant), Hindu 828 mgC3GE/kg of eggplant), Thai (39 mgC3GE/kg of eggplant), and higher than those reported in the Black Bell eggplant type [6], Tunisina, Buia, and L305 [44] raw, grill and boiled (50 to 90, 15 to 41, 31 to 155, and 17 to 96 mg D3R/ 100 g of dry matter, respectively).

### 2.9. Antioxidant Capacity

Currently, there are numerous methods to measure the antioxidant capacity of a food. In this study, the antioxidant capacity of the flours was measured by using three methods (DPPH, ABTS, and FRAP), using vitamin E analogue as reference (Trolox).

Determination of scavenging stable DPPH free radical is a quick way to evaluate the antioxidant activity of the extracts [45]. Table 5 shows the DPPH activity results of all four different samples. The range was between 9111 to 54,815 (μM Trolox equivalents/kg flour). Nino-Medina et al. [16] found higher results than the results of this study, which were 78,500 μM Trolox equivalents/kg on frozen, dried American eggplant type.

The ABTS (2,2′-azinobis-(3-ethylbenzothiazoline-6-sulfonic acid) assay is generated by the oxidation of the ABTS with potassium persulfate [46]. The results for ABTS assay ranged from 14,272 to 63,583 (μM Trolox equivalents/g flour). These results can be seen in Table 5. The results of this study were higher than those reported by Okmen et al. [47]. Their report was based on a study of total water soluble antioxidant activity of 26 eggplant (*Solanum melongena* L.) cultivars from Turkish with an antioxidant activity range from 2664 μM Trolox equivalents/kg to 8247 μM Trolox equivalents/kg.

The ferric reducing antioxidant power (FRAP) assay measures the ability of eggplant flour to reduce Fe^3+^/tripyridyltriazine complex to its ferrous form [48]. The results shown in Table 5 reveal a significant difference between μM Trolox equivalents/kg flour; the results ranged from 17,820 to 105,617 μM Trolox equivalents/kg flour. Results reported for eggplant extract [43] with different solvents (acetone, methanolic, and ethanolic) were in the range of 21,000 to 27,000 mg of quercetin equivalent/kg of extract.

In general terms, the results of antioxidant activity, such as the content of total phenols content and total flavonoids content, follow the following order T1 > T4 > T1 > T3. A highly significant difference was found between the samples; the sample treated with a pre-treatment of slicing/freezing before drying was the most affected sample, followed by the sample crushed before drying, as explained above in Section 2.5 and Section 2.6. Concellon et al. Reference [49] found that eggplant (American type) stored at 0 °C had a rapid degradation of antioxidant compounds. This behavior was described by other authors [49,50] as related to the antioxidant and phenolic content with the degree of browning of the eggplant. Eggplants generate a cellular disruption when being cut, with a loss of compartmentalization that allows contact between enzymes responsible for browning, such as polyphenoloxidase (PPO) and phenolic substrates [49,51,52,53]. Treatments such as mincing and freezing, and the time of exposure to air and light contribute to the generation of the browning of the eggplant, thus affecting both its content and antioxidant capacity.

## 3. Materials and Methods

### 3.1. Flour Preparation

The eggplants fruits used in this study did not had the quality requirements for exportation market (American type) and were purchased from local market in San Nicolas de los Garza County (Nuevo Leon, Mexico). The chemical composition of the eggplant was: Moisture 90%, ash 0.55%, protein 1.07%, fat 0.15%, 1.03% crude fiber, and carbohydrate 5.69%. The fruits (60 units, 25 kg) were washed and separated into four treatments. The fruits of treatment one (T1) were minced (Cyclone Sample mill-model 3010-030, UDY Corporation, Fort Collins, CO, USA) and dried in the drying oven (Model 630, Napco, Oregon, OR, USA) at 45 °C to 50 °C for 2 days. The fruits of treatment two (T2) were sliced and dried using the same condition of the first group. The fruits of treatment 3 (T3) were sliced, frozen, and dried in a tunnel dryer (Procmex Model LQ001, Procomm, Mexico) at 40 °C to 45 °C for 16 h. In the treatment four (T4) the fruits were sliced and dried with the same condition of the third group. The drying temperature range were based on the experience of Uthumporn et al. and Vega-Galvez et al. [4,9] in order to keep the content of phenolic compounds. To determine the drying time, the humidity content was measured as a preliminary result until a percentage below 15% (Mexican Standard NOM-247-SSA1-2008) [13] was obtained. Two drying methods were used, a drying oven (no air circulation and in total darkness, 15.5% humidity,) and a drying tunnel (air circulation, which passes through a set of resistance becomes dry air, 2.5% humidity). The tunnel design allows for the entrance of light, yet it was controlled. The flour was stored in a refrigerator at 4 °C prior to use. Table 6 describes the conditions of the treatments.

### 3.2. Proximate Composition 

Analyses were performed according to the Association of Official Analytical Chemistry [54]. Ash, moisture, and crude fiber content were evaluated gravimetrically (method AOAC 14.006, AOAC 925.15, and AOAC 962.09, respectively). The Goldfisch method (AOAC 920.36C) was used to determine the fat content. The protein content was measured using the Kjeldahl method (AOAC 930.29), and total carbohydrates were determined by difference.

### 3.3. Physicochemical Properties

In order to measure the potential of hydrogen (pH) and titratable acidity (TA), 10 mL of sample were diluted with 40 mL of distilled water; then, the pH was read. After that, samples were titrated with 0.1 M NaOH to a pH 8.2 (citric acid as predominant) using a Corning, 440 pH meter (Woburn, MA, USA) according to the Association of Official Analytical Chemist methods [55]. 

For color determination, a 1.5 mL spectrophotometric cuvette was filled with sample and color and was measured using a CR-20 Konica Minolta Color Reader (Tokyo, Japan). Chromatic parameters were obtained using CIELAB (L*, a*, b*) and CIELCH (L*, C*, *h*) color systems according to Commission Internationale De L′ecleirage [56]. L* defines Lightness (0 = black, 100 = white), a* indicates red (positive a*) or green value (negative a*) and b* indicates yellow (positive b*) or blue value (negative b*), C* (Chroma; saturation level of *h*), and *h* (hue angle: 0° = red, 90° = yellow, 180° = green, 270° = blue). Color view was obtained by using the online software ColorHexa, color converter using L*, a*, and b* values [57].

### 3.4. Functional Properties 

Water and oil holding capacity were determined according to the method described by Beuchat [58] with some modifications; 0.5 g of the sample were taken in 5 mL of distilled water (pH was adjusted to 7) or vegetable oil and mixed by vortexing (model V2H, Boeco, Hamburg, Germany) for 1 min. Then, it was centrifuged at 3000 rpm/30 min. The results were expressed in grams of water-oil retained per gram of sample. The measurements were carried out at room temperature.

For the emulsifying activity, the methods described by Yasumatsu et al. [59] and Zhao et al. [60] were used; 0.5 g of sample with 20 mL of distilled water were mixed in a vortex for 15 min and the pH was adjusted to 7. Vegetable oil was mixed in a relation 1:1 (20 mL) and homogenized (OMNI GLH model glh-01, OMNI International, Georgia, GE, USA) for 3 min at medium speed, and it was then centrifuged at 1300 rpm. The results were expressed as a percentage of the height of the emulsification layer with respect to the total liquid.

### 3.5. Preparation of the Eggplant Flour Extracts (EFE)

Dried powder (90 mg) was extracted with 5 mL of 80% methanol. The extraction was carried out at room temperature, using a magnetic stirrer. After 40 min, the solution was centrifuged for 5 min at 9500× *g* (10 °C). The supernatant was collected and stored under refrigerated conditions until it was used.

### 3.6. Total Phenols Content (TPC)

The total phenols content was determined by using the Folin-Ciocalteu method [61]. This was carried out by mixing 200 μL of the samples extract with 2.6 mL of distilled water, 200 μL of Folin-Ciocalteu reagent, and 2 mL of sodium carbonate solution (7%). After 120 min in the dark (incubation was at room temperature, 23 °C–25 °C), absorbance was measured at 730 nm. The total phenolic content was expressed as mg of chlorogenic acid equivalent (CAE) per 100 g of eggplant flour.

### 3.7. Total Flavonoids Content (TFC)

The total flavonoids content was measured using the Xiong et al. [62] method, with slight modifications. Briefly, 200 μL of the samples extract were mixed with 3.5 mL of distilled water and 150 μL of 5% NaNO_2_ solution. After 5 min, 150 μL of 10% AlCl_3_ solution were dissolved in distilled water, which was added. The mixture was incubated at room temperature for 5 min; then, 1 mL of 1 M NaOH was added and vortexed well for 5 s and left for 15 min. TFC was expressed as mg of catechins equivalent (CAE) per 100 g of eggplant flour.

### 3.8. Total Catechins Content (TCC) 

The total catechins content of the extract was determined by using the vanillin method [63] with some modifications. The sample extract (250 μL) was mixed with a solution of 1% vanillin (650 μL) and a solution of 25% H_2_SO_4_ (650 μL). After 15 min, the solution was incubated at 30 °C; absorbance was measured at 500 nm. TCC was expressed as mg of catechins equivalent (CAE) per gram of eggplant flour.

### 3.9. Total Anthocyanins (TAC)

The total anthocyanins content was evaluated according to Abdel-Aal and Hucl [64]. For the extraction of anthocyanins, 200 mg of maize flour was mixed with 10 mL of ethanol-HCl 1N (85:15 *v*/*v*, pH 1, 4 °C), purged for 30 s with argon and stirred for 30 min at 200 rpm. Afterwards, the sample was centrifuged at 7759× *g* (4 °C, 15 min) and finally, 3.5 mL of sample was measured at 535 nm. The content of was reported as milligrams of cyanidin-3-glucoside (C3G) per kilogram of flour (mgC3GE/kg) as follows: C = (A/ε) × (V/1000) × MW × (1/weight of sample) × 10^6^, where: C = concentration in mgC3GE/L, A = absorbance of sample, ε = molar absortivity (mgC3GE = 26,965 cm^−1^mol^−1^), V = volume of sample, and MW = molecular weight of C3G (449.2 g/mol).

### 3.10. Determination of Antioxidant Capacity

The electron-hydrogen donation ability of eggplant flour extract was measured by using DPPH, ABTS, and FRAP methods. The DPPH method was performed according to the method described by Tai et al. [65] with slight modifications. For this, 1.5 mL of 2 mg/L DPPH solution in methanol 80% and 50 μL of sample were mixed, and incubated at room temperature (23 °C–25 °C) in darkness for 30 min. The absorbance was measured at 517 nm against a blank. Results were expressed in micromoles of Trolox equivalents (μMTE)/g eggplant flour.

For the ABTS assay, the procedure followed the method used in previous assays [66,67] with a few modifications. The stock solutions included 2.6 mM potassium persulfate solution and 7.7 mM ABTS·+ solution; these solutions were mixed in equal quantities. After 12 h at room temperature in the darkness, the ABTS·+ solution was diluted with methanol 80% to obtain an absorbance of 1.000 units at 734 nm using the spectrophotometer. The eggplant flour extract (50 μL) was allowed to react with 1500 μL of ABTS·+ solution for 30 min in the dark; then, the absorbance was measured at 734 nm. Results are expressed in micromoles of Trolox equivalents (μMTE)/g eggplant flour. 

The ferric reducing-antioxidant capacity was measured according to the method described by Suárez et al. [68] with slight modifications. The working FRAP reagent was prepared with 5 mL of TPTZ (10 mM), 5 ml of FeCl_3_ (20 mM), and 50 mL of sodium acetate buffer (300 mM, pH = 3.6). Then, 50 μL of the sample extract were mixed with 1.5 mL of freshly working FRAP reagent. The FRAP assay was carried out at 37 °C in an incubator. The absorbance was measured at 595 nm and the results were expressed in micromoles of Trolox equivalents (μMTE)/g eggplant flour.

### 3.11. Statistical Analysis 

Data from the three replicated experiments were analyzed to determine whether the variances were statistically homogeneous, and the results were expressed as means ± SD. Statistical comparisons were made by one-way analysis of variance (ANOVA) followed by a Tukey’s test using SPSS 17 Software. Difference between means were considered significant at *p* < 0.05. 

## 4. Conclusions

All the eggplant flours showed the same trend regarding their antioxidant capacity and phenolic content in the order T2 > T4 > T1 > T3. The freezing of eggplant was found to have a negative effect on functional and antioxidant properties. With respect to their nutritional composition, the flours did not change in their crude fiber, protein, and fat contents. In general terms, the T2 flour is a potential ingredient for the preparation of foods with functional properties since it is rich in phenolic compounds and antioxidants.

## Figures and Tables

**Table 1:** Nutritional components of different obtained flours.

Component (%)	T1	T2	T3	T4
**Moisture**	5.26 ± 0.4 ^b^	8.57 ± 0.26 ^a^	4.55 ± 0.32 ^c^	1.57 ± 0.09 ^d^
**Ash**	6.47 ± 0.38 ^b^	7.31 ± 0.03 ^a^	7.31 ± 0.08 ^a^	6.53 ± 0.25 ^b^
**Fat**	1.79 ± 0.07 ^a^	1.75 ± 0.03 ^a^	1.73 ± 0.0 ^a^	1.73 ± 0.02 ^a^
**Protein**	12.57 ± 0.39 ^a^	12.5 ± 0.45 ^a^	12.68 ± 0.29 ^a^	12.77 ± 0.24 ^a^
**Crude Fiber**	12.74 ± 0.37 ^a^	12.32 ± 0.43 ^a^	11.8 ± 0.59 ^a^	12.17 ± 0.92 ^a^
**Carbohydrates ^+^**	61.17 ± 0.7 ^b^	57.54 ± 0.52 ^c^	61.92 ± 0.18 ^b^	65.22 ± 1.22 ^a^

Eggplant minced and dried in a drying oven (T1), sliced eggplant dried in a drying oven (T2), sliced eggplant frozen and dried in a tunnel dryer (T3), and sliced eggplant dried in a tunnel dryer (T4). Average values with three replicates ± standard deviations, of three different lots. Mean values labeled with a different letter in the same file are significantly different (*p* < 0.05). ^+^ Carbohydrates (%) = 100 − (% moisture + % ash + % fat + % protein + % crude fiber).

**Table 2:** Physicochemical parameters of different eggplant flours.

Flour	pH	TA (%)	Chromatic Properties					
			L*	a*	b*	C*	*h*	View
**T1**	3.89 ± 0.04 ^b^	0.47 ± 0.004 ^a^	52.50 ± 0.14 ^c^	9.65 ± 0.49 ^a^	21.65 ± 0.49 ^a^	23.60 ± 0.65 ^a^	65.98 ± 0.60 ^b^	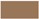
**T2**	3.97 ± 0.02 ^b^	0.47 ± 0.004 ^a^	52.55 ± 0.49 ^c^	9.25 ± 0.63 ^ab^	21.05 ± 0.49 ^a^	22.99 ± 0.71 ^ab^	66.29 ± 0.95 ^b^	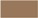
**T3**	4.19 ± 0.01 ^a^	0.46 ± 0.009 ^a^	57.40 ± 0.42 ^b^	6.90 ± 0.56 ^b^	20.15 ± 0.21 ^a^	21.30 ± 0.01 ^b^	71.10 ± 1.62 ^b^	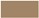
**T4**	4.14 ± 0.03 ^a^	0.46 ± 0.004 ^a^	64.60 ± 0.42 ^a^	4.55 ± 0.07 ^c^	20.60 ± 0.14 ^a^	21.09 ± 0.16 ^b^	77.54 ± 0.10 ^a^	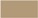

Potential of hydrogen (pH), titratable acidity (TA) and chromatic properties (L*, a*, b*, C*, *h*) of eggplant flours. Values are the average of three replicates ± standard deviations, of three different lots. Mean values labeled with a different letter in the same column are significantly different (*p* < 0.05).

**Table 3:** Functional properties of eggplant flours.

Flour	WHC (g Water/g Flour DW)	OHC (g Oil/ g Flour DW)	EC (%)
**T1**	1.28 ± 0.1 ^b^	2.13 ± 0.26 ^d^	25.00 ± 0.10 ^c^
**T2**	1.61 ± 0.16 ^b^	4.49 ± 0.59 ^b^	37.33 ± 0.57 ^a^
**T3**	1.40 ± 0.25 ^b^	3.79 ± 0.16 ^c^	34.50 ± 0.50 ^b^
**T4**	2.08 ± 0.13 ^a^	5.22 ± 0.11 ^a^	37.83 ± 0.28 ^a^

Water holding capacity (WHC), oil holding capacity (OHC) and emulsion capacity (EC) of eggplant flours. DW = dried weight. Values are the average of three replicates ± standard deviations, of three different lots. Mean values labeled with a different letter in the same column are significantly different (*p* < 0.05).

**Table 4:** Phenolic compounds of eggplant flours.

Flour	TPC (mgCAE/kg Flour DW)	TFC (mgCatE/kg Flour DW)	TCC (mgCatE/kg Flour DW)	TAC (mgC3GE/kg Flour DW)
**T1**	8211 ± 452 ^c^	2060 ± 396 ^c^	3022 ± 330 ^a^	1612 ± 44 ^a^
**T2**	18,227 ± 442 ^a^	15,753 ± 1027 ^a^	1461 ± 176 ^c^	679 ± 12 ^b^
**T3**	4183 ± 123 ^d^	1473 ± 188 ^c^	1240 ± 206 ^c^	230 ± 13 ^d^
**T4**	10,866 ± 673 ^b^	10,300 ± 467 ^b^	2307 ± 145 ^b^	519 ± 10 ^c^

Total phenols content (TPC), total flavonoid content (TFC) and condensed tannin content (CTC). mgCAE = milligrams of chlorogenic acid equivalents, mgCatE = milligrams of catechin equivalents, mgC3G = milligrams of cyanidin-3-glucoside, DW = dried weight. Values are the average of three replicates ± standard deviations, of three different lots. Mean values labeled with a different letter in the same column are significantly different (*p* < 0.05).

**Table 5:** Antioxidant activity of eggplant flours.

Flour	ABTS (μMTE/kg Flour DW)	DPPH (μMTE/kg Flour DW)	FRAP (μMTE/kg Flour DW)
**T1**	25,484 ± 1166 ^c^	15,160 ± 142 ^c^	29,534 ± 315 ^c^
**T2**	63,583 ± 1689 ^a^	54,815 ± 2447 ^a^	105,617 ± 3917 ^a^
**T3**	14,272 ± 433 ^d^	9111 ± 160 ^d^	17,820 ± 587 ^d^
**T4**	43,205 ± 673 ^b^	43,167 ± 3611 ^b^	75,361 ± 773 ^b^

μMTE = micromoles Trolox equivalents. DW = dried weight. Values are the average of three replicates ± standard deviations of three different lots. Mean values labeled with a different letter in the same column are significantly different (*p* < 0.05).

**Table 6:** Pre-treatments and drying method for obtaining of eggplant flours.

Flour	Pre-Treatments	Equipment
**T1**	Mincing	Drying oven
**T2**	Cutting in slices	Drying oven
**T3**	Cutting in slices and freezing	Tunnel dryer
**T4**	Cutting in slices	Tunnel dryer
